# Exploring Aluminum Tolerance Mechanisms in Plants with Reference to Rice and *Arabidopsis*: A Comprehensive Review of Genetic, Metabolic, and Physiological Adaptations in Acidic Soils

**DOI:** 10.3390/plants13131760

**Published:** 2024-06-25

**Authors:** Nilakshi Chakraborty, Abir Das, Sayan Pal, Soumita Roy, Sudipta Kumar Sil, Malay Kumar Adak, Mirza Hassanzamman

**Affiliations:** 1 Plant Physiology and Molecular Biology Research Unit, Department of Botany, University of Kalyani, Kalyani 741235, West Bengal, India; 2 Department of Botany, University of Gour Banga, Malda 732103, West Bengal, India; 3 Department of Agronomy, Faculty of Agriculture, Shar-e-Bangla Agricultural University, Dhaka 1207, Bangladesh; 4 Kyung Hee University, 26 Kyungheedae-ro, Dongdaemun-gu, Seoul 02447, Republic of Korea

**Keywords:** toxic metals, environmental pollution, antioxidant defense, organic acid exudation, programmed cell death, vacuolar processing enzymes

## Abstract

Aluminum (Al) makes up a third of the Earth’s crust and is a widespread toxic contaminant, particularly in acidic soils. It impacts crops at multiple levels, from cellular to whole plant systems. This review delves into Al’s reactivity, including its cellular transport, involvement in oxidative redox reactions, and development of specific metabolites, as well as the influence of genes on the production of membrane channels and transporters, alongside its role in triggering senescence. It discusses the involvement of channel proteins in calcium influx, vacuolar proton pumping, the suppression of mitochondrial respiration, and the initiation of programmed cell death. At the cellular nucleus level, the effects of Al on gene regulation through alterations in nucleic acid modifications, such as methylation and histone acetylation, are examined. In addition, this review outlines the pathways of Al-induced metabolic disruption, specifically citric acid metabolism, the regulation of proton excretion, the induction of specific transcription factors, the modulation of Al-responsive proteins, changes in citrate and nucleotide glucose transporters, and overall metal detoxification pathways in tolerant genotypes. It also considers the expression of phenolic oxidases in response to oxidative stress, their regulatory feedback on mitochondrial cytochrome proteins, and their consequences on root development. Ultimately, this review focuses on the selective metabolic pathways that facilitate Al exclusion and tolerance, emphasizing compartmentalization, antioxidative defense mechanisms, and the control of programmed cell death to manage metal toxicity.

## 1. Introduction

Acidic soils, comprising nearly 40% of the Earth’s crust, include about 50% of arable land. These soils can support certain crop species, though they often require altered physiological responses. In acidic soils, Al is the most abundant element, significantly impacting rice cultivation, which occupies 13% of acidic land [1]. Al exists in various forms, including aluminum silicate and sulfate, and can dissolve into active trivalent Al ions (Al^3+^), particularly under low-pH conditions in the rhizosphere. Dominant forms like aluminum hydroxide cations [Al(OH)_2_^+^, Al(OH)_3_^+^] can cause oxidative stress. Both inorganic and organic residues in the rhizosphere contribute to Al release into acidic water bodies [2]. Exchangeable Al^3+^ binds with silicates, clays, and other residues, affecting mineral availability to plants. High Al concentrations, depending on soil pH, can damage root systems.

In acidic soil, roots are impaired with membrane permeability, hydraulic conductivity, mineral acquisition, and the replacement of other nutrients, which are collectively responsible for plant growth and productivity [3]. Al toxicity involves inducible gene expression and two primary stress response mechanisms. Bio-exclusion of Al involves both internal and external mechanisms for Al, and those essentially need organic acid-based ligand formation.

The effects of Al accumulation are more notable in the root apex, root cap, and extended permanent regions of roots, where altered carbohydrate metabolism is directly related to metal uptake. In Al-tolerant genotypes, the uptake of metal may not be related to impeded ion uptake for nitrate (NO_3_^−^)-like ions [4]. In cereals, the inhibition of such ion uptake is reversed by ammonium (NH_4_^+^) treatment, which suggests the co-transport of Al with other cations. Still, the interaction of Al with NO_3_^−^ is a possible clue for mineral imbalances under acidic soil. The increase in amino acids under Al treatment is also assumed for an enhanced degradation of proteins in the root zone. Likewise, a proportion of amines (asparagines, glutamine) are the product of Al-induced protein hydrolysis or oxidation, and thereby, the accumulation of free amino acids serves as a bio-indicator of acidic soil [5]. On the other hand, uptake of ions is reduced due to competition with Al according to the pH of the solution. Thus, reduction in NO_3_^−^ absorption releases more protons (H^+^) into the rhizosphere that otherwise favor acidic soil pH and more Al absorption. A decline in NO_3_^−^ uptake influences signal(s) for rhizosphere secretion by Al-sensitive species to release some special metabolites. Thus, NO_3_^−^ uptake would be a preliminary symptom of Al toxicity within a short period of application of the metal, particularly in tolerant plants.

Toxicity to Al is another path irrespective of tolerant and susceptible species where different chemicals may function as elicitors of defense mechanisms. In relation to crop species grown in acidic soil, there are various mechanisms of Al tolerance [6]. Few predominant chemical species (malate, oxaloacetate, citrate, and a few other organic acids) are released into the rhizosphere and act as Al chelators. This is an extracellular strategy of Al avoidance by specific reactions in different tolerant genotypes with variations in the sequestration of metal [7]. In intracellular pathways, the same strategy for metal chelation is operative, where H^+^ pump-mediated cytoplasmic and vacuolar acidity is accomplished. Thus, Al sensitivity covers acidolysis of the cytoskeleton and biomolecules to different degrees depending on reactions in non-tolerant species. The subsequent imprisonment of the metal into non-cellular or apoplastic spaces also sets up another tolerance mechanism. Moreover, Al toxicity also leads to a change in the membrane potential, which is often correlated with the membrane surface binding capacities of other cations [8]. A change in plasma membrane potential induced by Al-mediated depolarization can also regulate calcium ion (Ca^2+^) uptake, influencing the growth of the plants. The effect of cytoplasmic Ca^2+^ concentration directly regulates many processes of cell metabolism where Al injury is responsible. Al-dependent flux of Ca^2+^ from extracellular spaces regulates the inhibitions of root elongation, which may be reversed by acidification of the soil [9]. Therefore, the cellular pH of the roots is the determining factor for the availability of some signaling molecules (e.g., Ca^2+^) for the interaction of Al in soil. A pH-dependent synthesis of complex polysaccharides (like 1-3-glucan) in the roots influences metabolism of calcium, where the roles of calmodulin (CaM)-like binding proteins are important [10].

Despite the significant body of literature describing the toxicity of Al in plants, little is deciphered about specific ion effects like oxidative stress. It was reported earlier that besides water relations and other cellular anomalies induced by Al as a metal in general, changes in redox are the prime key to Al vulnerability. The accumulation of reactive oxygen species (ROS) and, thereby, the induction of antioxidation machineries are the keys to Al tolerance [11]. A change in cellular redox, inducing oxidative stress, is the initiation of different chemical reactions like the peroxidation of lipids, the carbonylation of protein, the tautomerization of nucleic acids, all of which are processes of the degeneration of plants under Al toxicity. Furthermore, Al-induced inhibitions of cellular respiration and adenosine triphosphate (ATP) depletion can compel the plants to bypass the electron flow from the electron transport chain (both in chloroplasts and mitochondria) into molecular oxygen. At the cellular level, in roots, the depletion of energy-utilizing pathways and the dysfunction of the organic acid cycle in the mitochondria lead to the loss of viability of roots [12]. Thus, oxidative stress is set as the key underlying mechanism of Al toxicity where interference from other redox metals (iron, chromium, zinc) in the tissue is complementary to the degenerative process of toxicity. More insights are yet to be known for ROS production through other pathways linked to cellular reactions as major determining factors for root growth inhibition under Al contamination.

This review aims to thoroughly understand the Al resistance mechanisms in tolerant genotypes and other species in acidic soils. It emphasizes the need to develop cultivars or identify specific traits to support adaptation to Al-rich acidic soils. Understanding increased Al^3+^ sensitivity is crucial due to the constraints of food production in specific agro-climatic zones; it is imperative to understand the tolerance mechanisms of crops for breeding purposes. Metal detoxification primarily relies on exclusion mechanisms, with organic acids forming stable complexes with Al^3+^ [13]. Genetic studies on rice, a crop highly sensitive to acidic soils, have identified several gene families (*ALMT*, *MATE*, *ABC*) and transcription factors regulatinggenes (*ART1*, *ASR5*, *OsWRKY22*) that are crucial for Al exclusion and tolerance [14]. This review also reveals the importance of omics technologies like genomics, proteomics, and metabolomics to understand the complex traits in crops under Al toxicity using intradisciplinary approaches in crop science. It may cover the generation of metabolites under metal stress with their qualitative and quantitative distribution, biosynthesis, catabolism, and the functional network in combination for tolerance. It also finally discusses the development of selective markers based on specific metabolomes and their roles in metal tolerance in plants through gene overexpression and chemical elicitation.

## 2. Aluminum Bioavailability in Different Forms to Sensitize the Plants

**Figure 1:**
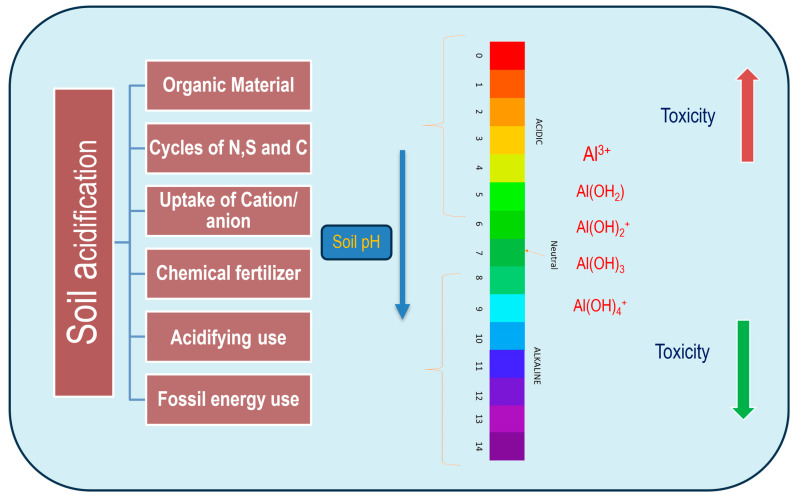
Illustration of soil acidification’s impact on Al chemistry, highlighting its transformation into various inorganic forms. Al reactivity varies with solubility, heavily influenced by ionic changes. Different Al species are formed based on acidic pH levels, with Al ions (Al^3+^) being the most predominant in acidic conditions, forming stable complexes with sulfate ions (SO_4_^2−^), hydroxide (OH^−^), phosphate ions (PO_4_^2−^), and silicon (Si). As pH increases, aluminum toxicity diminishes, leading to diverse complexes like aluminum hydroxide ions [Al(OH)_3_, [Al(OH)^4^]^−^, Al(H_2_O)_6_^3+^] and other insoluble hydroxides. Al^3+^ actively enters plant roots in environments with a pH lower than 5. At neutral pH (~7), Al(OH)_3_ forms, characterized by higher insolubility and non-toxicity. In environments with a pH greater than 7, aluminate [Al(OH)^4^]^−^ or aluminate anion species occur, often complexing with other molecular species such as AlO_4_Al_12_(OH)_24_(H_2_O)_12_^7+^.

Highly complex effluents from industrial outlets composed of different Al compounds are present in varying quantities and are responsible for toxicity in acidic soil. In soil with pH < 4, increasing crop susceptibility to metal toxicity due to a high concentration of Al can cause soil acidification, which is more targeted towards failure of crop productivity [15]. In spite of this, soil acidification is achieved by other agronomic factors through the accumulation of basic cations: K^+^, Na^+^, Mg^2+^, Ca^2+^, etc. Al is the major component, covering >7% of metallic elements in the Earth’s crust after silicon, yet showing no significant roles in plant growth and development [16]. Al is precipitated in soil from major ores like aluminosilicate that undergo initial solubilization (at pH < 5.0), resolving in an eventual phytotoxic form. Trivalent aluminum ions can also accompany different hydroxides of metal like aluminum hydroxide cations like Al(OH)^2+^, Al(OH)_3_, and [Al(OH)^4^]^−^ in partial to full solubilization for plants’ absorption. This is based on a gradual depletion of soil pH that corresponds to a better solubility of Al salts with different valences (Figure 1). Out of the different valences, Al^3+^ is the most toxic and stress-producing form, particularly when distributed through apoplastic and symplastic spaces in roots. Plant species irrespective of taxa when subjected to intense and prolonged durations of Al exposure undergo variable levels of modification through intra- and intercellular components [17]. Physiologically, these cover changes in cell wall components, modification of transport processes across the membrane, disruption of cytoskeletons, altered cell signaling energy-yielding metabolism, and, finally, the mutation of nuclear material [18]. Notably, plants that respond to Al^3+^ toxicity are distinctly categorized as either tolerant or sensitive species. Thereby, a search is underway for the tolerance mechanisms of plants in acidic soil when plant growth is vulnerable at physiological and genetic levels. The most predominant line of investigation is based on the absorption of Al through the root system, which changes root physiology and the biochemistry and molecular biology of cells. The second line of research is based on an analysis of induced oxidative stress with specific Al^3+^ ions at the cellular level [19]. Different oxidative metabolites, their varying expression through tolerant and sensitive cultivars, and regulation through biochemical reactions are the major domains of Al-induced oxidative stress in research. This is more intricate than other findings, where partially known or hypothetical ligands are reported to form complex compounds with free Al in the cytosol [20]. In this view, biosynthetic and catabolic genes are under study, concerning tolerant cultivars. Therefore, the acquired information may be relevant for the application of molecular breeding and biotechnological devices for the development of tolerant genotypes against Al toxicity.

## 3. Aluminum-Induced Oxidative Stress and Metabolic Alterations

Industrial processes and human activities are primary contributors to the dispersal of toxic forms of Al in the Earth’s crust. These activities release various toxic molecular forms of Al, posing significant threats to both the environment and plants’ lives. Sources of these toxic forms include the processing of ores, the use of fertilizers and herbicides, and the disposal of solid wastes such as sewage sludge and alloy emissions from different industries [21]. A key indicator of Al toxicity in plant cells is the generation of ROS. In particular, roots are highly sensitive to Al exposure, leading to the production of various ROS. This generation of ROS occurs not only in the cytosol but also within cellular organelles such as chloroplasts, mitochondria, and peroxisomes, which are rich in electron transport chains in cells [22]. In the nucleus, peroxidation of the membrane can initiate cascades of free radical generation, including hydroperoxides and peroxy radicals, which interact with nucleotides like macromolecules. Research has identified several genes activated in the nucleus that act upon tolerance mechanisms through cytoplasmic and organellar compartments. Key genes include *ADP-ribosyltransferase 1*, *ferric reductase defective like 4*, *acetolactate synthase 1*, and *Nramp family 1* (*ART1*, *FRDL4*, *ALS1*, *NRAT1*), along with various antioxidants and proteins involved in the ascorbate (AsA)–glutathione (GSH) cycle [23].

Mitochondria can also be significant sources of ROS, especially where malate and organic acid transporters facilitate the entry of Al ion (Al^3+^). The inter-membrane spaces, housing the electron transport chain, are prone to ROS generation, particularly hydroxyl radicals, resulting in oxidative decarboxylation for DNA. The redox status is crucial for understanding the metabolic impact of Al on tissues, with pyridine nucleotides (NADH/NAD^+^, NADPH/NADP^+^, FADH_2_/FAD^+^) playing a key role in signaling through ROS [24]. These ROS trigger various harmful reactions including lipid peroxidation, protein carbonylation, and the formation of bulky nucleotide adducts. The dual roles of ROS as critical signaling residues as well as in inducing oxidative stress are more contextual for signaling against Al ions (Al^3+^). Root tips affected with Al^3+^ are demonstrated with detoxification mechanisms through cell wall modification. ROS and their exposure on the outer surface of the plasma membrane induce a complex network of wall-modifying enzymes [25]. There are a few noticeable overexpressed proteins like expansions, xyloglucan endo-transglucosylase, and pectin acetyl esterase, the latter of which is responsible for cell wall elasticity as well as crosslinking with secondary deposition. ROS, particularly hydrogen peroxide (H_2_O_2_), are the most important inducers in the reaction where Al^3+^ is chelated with complex carbohydrate residues. The formation of superoxide radicals on apoplastic and membrane-bound NAD(P)H oxidase (NOX) and respiratory burst oxidase homolog (RBOH) is the most important due to their activities in signaling processes. A number of enzymes like superoxide dismutase, xanthine oxidase, and, finally, a few classes III wall-bound peroxidases are important for different ROS/free radicals in sensitization to Al. In the presence of Al^3+^, the crosslinking of cell wall residues, mostly xyloglucans, increases the simultaneous formation of ROS and the overexpression of variants of peroxidases. ROS on the cell wall and in apoplastic spaces are protruded with membrane transporters like nod26-like intrinsic protein (NIP), *Halimione portulacoides* plasma membrane amino acid transporter 1(HmPALT1), and nitrate transporter 1 (NRAT1), with simultaneous restriction of Al^3+^ entry [26]. In the latter stages of metal-ion-induced toxicity in the cytosol, ROS signal membrane transporters to overexpress organic acid biosynthesis. A few phenolic residues are also induced to chelate Al^3+^ in the rhizosphere to prevent the entry of ions through the root system. The path of ROS development under Al^3+^ toxicity is quite consistent with other external stimuli. The intricate control mechanism employing RBOH happens to be most useful in metal-affected tissues for the transmission of signals following its transduction [27]. Additionally, NOX-derived ROS are also accompanied by a number of apoplast-harboring proteins, mostly peroxidases (PRXs), polyamine oxidases (PAOs), quinone reductase, and other metal-bearing anime oxidases. An oxidative signal, mainly through altered mitochondrial electron pathways, also promotes ROS generation [28]. Finally, within the nuclear membrane, ROS exposure activates gene encoding for antioxidative cascades for the regulation of oxidative stress. The translocation of ROS as a signal through the cytosol can also induce a number of membrane-bound transporters like *Oryza sativa* L.-activated malate transporter 1 (*Os*ALS1) and vacuolar amino acid transporter 1 (VALT1) for conjugations of Al^3+^ as well as direct detoxification with non-thiol residues and phytochelatin [29]. ROS produced by herbicides and pesticides, like xenobiotics, may function in reliable biomarking for specific plant species. The metals inserted within the xenobiotics have a distinct path to be detoxicated in the plant system, initially by the spatio-temporal transitory accumulation of free radicals (e.g., H_2_O_2_) that may act in the evocation of signals. This is followed by the compartmentalization of metal in cytosolic or even apoplastic spaces in conjugation with glutathione-like compounds [30]. Elicitors like melatonin are effective in the chelation of pesticide-like residues following transport into vacuoles by ATP-binding cassette (ABC) transporters, as found in the detoxification of Al^3+^.Therefore, ROS sensing and signal transduction would be dependent on the type of stimuli and plants’ genotypic potential to minimize their effects, either by direct sequestering or by antioxidation.

Among the plethora of free radicals, H_2_O_2_ stands out for its dual role in signaling, potentially leading to either beneficial or detrimental outcomes in the context of Al toxicity. The synthesis of macromolecules such as lignin, which relies on peroxidase activity on H_2_O_2_, is an example of an immediate response to Al stress. Oxidative stress, indicated by the accumulation of H_2_O_2_, fuels the peroxidase activity that is necessary for lignin biosynthesis [31]. Concurrently, plants exercise a defense system through a combination of both enzymatic and non-enzymatic antioxidants to mitigate a H_2_O_2_-induced peroxidation reaction.

**Figure 2:**
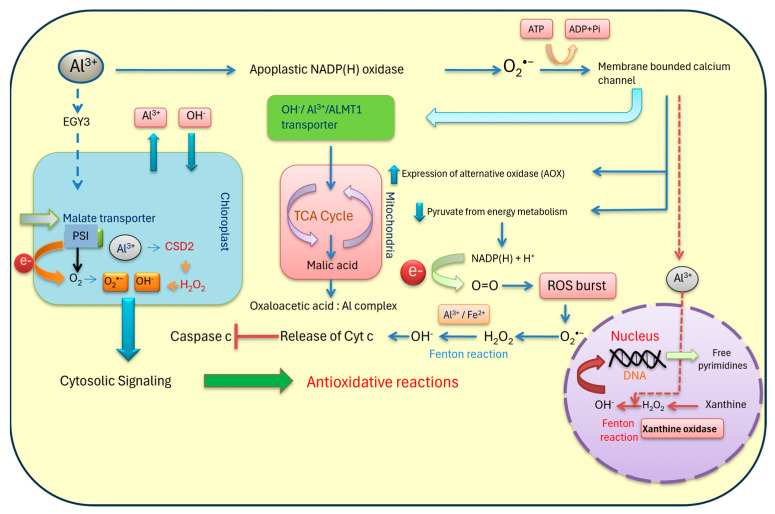
Integrated approach for ROS-mediated cellular tolerance of plants to Al toxicity. Al shares a common path for organic acid transporters (OA: Al^3+^) where the ions diffuse inside the cytosol and interact with the mitochondrial oxidative cycle. Malate dehydrogenase (MDH) and succinate dehydrogenase (SDH) are the major enzymatic sources where nicotinamide adenine dinucleotide [NAD(H) + H^+^] contributes electrons from O_2_ into superoxide anions (O_2_^•−^) and hydrogen peroxide (H_2_O_2_). In sensitive cultivars, H_2_O_2_ leads the formation of hydroxide ions (OH^−^) with the Fenton reaction. On the other hand, the nucleus is activated in nuclear membrane oxidation, which also produces free radicals and the induction of other genes: *angiotensin II receptor type 1*, *nitrate transporter 1* (*ATR1*, *NRT1*, etc.). Al-induced release of cytochrome c inhibits caspase-like activity, forwarding cell apoptosis. Chloroplasts’ reactions in another form can also induce ROS formation through the introduction of ethylene-dependent gravitropism-deficient and yellow-green light 3 (*EGY3*)-like metalloproteases that are efficient in the production of H_2_O_2_, which is retrograded in downstream signaling. The *EGY3* can also induce malate transporters, where Al^3+^ can invade PSI. The latter is induced to develop O_2_^•−^/OH^−^-like free radicals and are engaged in chloroplast membrane oxidation. Al^3+^ can also induce a chain of oxidative reactions where nucleotides are released freely by the action of xanthine oxidase-like activities.

In chloroplasts, Al toxicity triggers ROS production through mechanisms involving the electron transport chain in Photosystems I and II. Various ROS forms are generated, including singlet oxygen, superoxide, hydrogen peroxide, and hydroxyl radicals. The uptake of Al^3+^ induces the expression of certain genes in the chloroplast genome, which include membrane-bound ATP-independent proteases that play a significant role in Al tolerance mechanisms [32]. The mechanisms of Al tolerance are complex, involving the synergistic contributions of metal compartmentalization, chloroplast fluorescence, mitochondrial oxidative reactions, and peroxisomal carbon oxidation (Figure 2). Therefore, Al tolerance is a multifaceted approach that essentially underscores the intricate networks of responses of plants against oxidative stress.

Al profoundly impacts root growth and extends its detrimental effects to other plant organs. Many species show signs of toxicity at different organizational levels, from morphological changes to nuclear alterations, largely due to their oxidative sensitivity to the metal [33]. The solubilization of Al^3+^ in the rhizosphere, a result of soil acidification, leads to the formation of various chemical species of Al. This complicates agricultural practices in such soils, especially with the co-presence of other metals like ferrous ion (Fe^2+^) and various Al salt intermediates. In regions like the root meristem, endodermis, and cortical tissues, exposure to Al^3+^ causes lipid peroxidation and the accumulation of ROS, as demonstrated in maize cultivars. This results in significant inhibition of tissue elongation, highlighting the root as the primarily affected organ [34]. Al toxicity triggers oxidative stress, which alters cellular and membrane integrity by disrupting redox balance within cells. Different ROS types can initiate peroxidation reactions on macromolecules such as lipids, proteins, and nucleic acids, compromising cell viability.

Although Al is not a transition metal and does not directly catalyze redox reactions, it can still contribute to the oxidation of biomolecules, leading to oxidative stress. This often involves Al forming electrostatic bonds with biomolecular groups such as PO_4_^3−^ or COO^−^ on plasma membrane pectins [35]. The formation of callose within cell walls serves as an adaptive strategy to mitigate free radical damage. Transition metals, in conjunction with Al, contribute to the pool of ROS. Excessive Al on the membrane surface can increase the uptake of redox-active iron (Fe) into the rhizosphere, creating a vicious cycle of ROS production in plant tissues [36]. Crop species, including barley, rice, and gram, suffer from Al toxicity at their root apices, leading to growth inhibition due to lipoxygenase activity. In maize, factors such as early senescence and altered water relations, alongside oxidative stress, amplify the detrimental effects [37].

**Figure 3:**
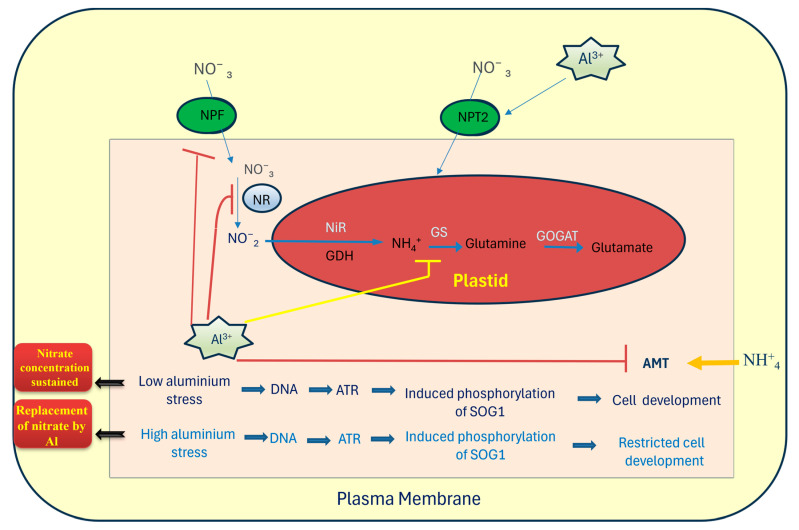
Depiction of NH_4_^+^ and NO_3_^−^ absorption by Al at root–soil interfaces. NH_4_^+^ lowers the rhizospheric pH, increasing the inhibition of metal uptake in plant roots due to competitive inhibition between Al^3+^ and H^+^. Conversely, rhizospheric pH becomes more alkaline with NO_3_^+^ accumulation, aiding in the desorption of metals into the rhizosphere but enhancing their transport into roots. Nitrate addition increases the negative electrical potential on the root surface, facilitating the conversion of NH_4_^+^ to NO_3_^−^. Excess NH_4_^+^/H^+^ can displace soluble NO_3_^−^ in roots. In sensitive cultivars, NH_4_^+^ can influence the binding of ataxia telangiectasia mutated (ATM) and ataxia telangiectasia mutated rad3-related (ATR) with suppressor of gamma response 1 (SOG1) for DNA damage recognition, leading to arrested cell growth in roots under high Al stress. The caption also references key enzymes and transporters involved in nitrogen metabolism: nitrite reductase (NiR), nitrate reductase (NR), glutamine synthase (GS), glutamate dehydrogenase (GDH), glutamine oxoglutarate aminotransferase (GOGAT), nitrate transporter 1/peptide transporter family (NPF), and nitrate transporter 2 family (NRT2).

In acidic soils, Al accumulation can inhibit the function of cellular proton-ATPase pumps (H^+^/ATPase), leading to the efflux of potassium and other essential elements from the membrane. The impact of Al-induced oxidative stress varies among crop species, depending on their genetic makeup. Metabolomic responses in susceptible species are critical in acidic conditions, where micromolar concentrations of Al^3+^ have specific toxic effects, impairing water and nutrient uptake. Combating soil acidification, a mounting global concern, involves selecting Al-tolerant genotypes and identifying and cloning specific genes related to Al tolerance [38]. A comprehensive understanding of cellular responses and nuclear regulatory mechanisms is vital for developing Al-tolerant crop lines. Metabolomics, reflecting changes in metabolite expression triggered by various Al species, needs further investigation. The interaction between nitrate and Al in root uptake significantly affects primary metabolomes [39]. Gene expression changes involve transcription factors like suppressor of gamma response 1 (SOG1), as well as DNA damage signaling proteins such as Ataxia-telangiectasia-mutated, and Rad3-related (ATR). In acidic environments where Al toxicity is prevalent, a higher ratio of ammonium ions to nitrate ions (NH_4_^+^ to NO_3_^−^) exists. Plants resistant to Al exhibit a preference for NH_4_^+^ over NO_3_^−^, facilitating better solubilization of Al in the rhizosphere (Figure 3).

## 4. Metabolome Induction to Aluminum Responses in Plants

**Figure 4:**
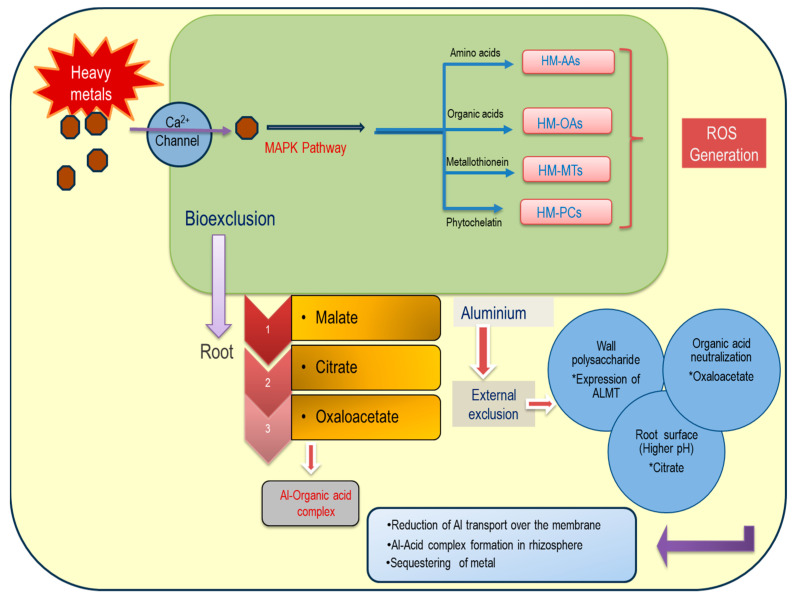
Illustration of Al tolerance mechanisms in plants through bio-exclusion during stress. The plant’s response to Al stress includes the formation of Al–organic acid (* oxaloacetate and * citrate as predominat residues) complexes, leading to increased alkalization of the root surface. This process involves the induction of Al-malate transporters (* ALMT), activation of proton ATPases, and alterations in cell wall polysaccharide composition. These adaptations aim to minimize Al accumulation within cells, thereby mitigating oxidative damages through enhanced antioxidation pathways.

The response to Al toxicity in plants involves two primary pathways: exclusion and oxidative tolerance [40]. Exclusion mechanisms involve several channel proteins on cell membranes and tonoplasts, including anion transporters (like malate/hydroxide ion), that facilitate Al sequestration into vacuoles or apoplastic spaces [41]. The solubility of various organic acid anions like citrate, lactate, oxalate, and succinate in acidic environments also plays a role [42]. Carbohydrate biosynthesis, particularly for cell wall components such as pectin, acidic heteropolysaccharides, D-galacturonic acid, and glycoside-containing residues like rhamnose and galactose, is crucial. Pectin, a prevalent residue for Al^3+^ chelation, is recycled from primary metabolites. Monosaccharides like galactose and cellobiose bind Al^3+^ on the cell wall, while lignin, among the heteropolysaccharides, is pivotal for covalent Al binding, influencing metal compartmentalization [43]. Al-tolerant plant genotypes exhibit enhanced lignin biosynthesis, supporting anatomical adaptations like vascular lumen thickening under metal stress [44]. Lignin and phenolics like coumarins, derived from the phenylpropanoid or shikimic acid pool, are significant [45]. Key enzymes overexpressed under Al toxicity include phenylalanine ammonia lyase (PAL) and coumarate CoA ligase [46], especially in rice cultivars. In plants, a multistep bio-exclusion of Al^3+^ from root cells is understood specifically for tolerant species where ions are entered through the symplast of tissues crossing the cell wall (Figure 4).

Al tolerance also involves malate transporters (AlMTs) in wheat and *Arabidopsis*, essential for Al exclusion [47]. Increased cytoplasmic Ca^2+^ is another aspect of Al tolerance, affecting cell division inhibition in roots [48]. In wheat root apices, Al toxicity inhibits phospholipase C (PLC), affecting downstream phosphatidyl inositol 4, 5 bisphosphate (PIP2) signaling. PIP2 and its associated genes, along with actin proteins, play roles in Ca^2+^ signaling under Al stress [49]. The source of Ca^2+^ in Al toxicity is still uncertain, but it influences downstream metabolism and plant sensitivity [50].

Al toxicity impacts secondary metabolite metabolism, particularly in cell walls and phloem tissue [51]. Genes for transporters like glucosyl transferase and antioxidative secondary metabolites like hydroxy ferulic acids have been identified [52]. Hormonal biosynthesis genes and metal chelators are also implicated in various secondary metabolite pathways [53]. Metabolites overexpressed under Al toxicity include carotenoid and indole alkaloid biosynthesis and pinene degradation, indicating distinct mechanisms for Al exclusion and tolerance. Adenosine triphosphate binding box (ABC) proteins play roles in energy-dependent Al transport and ATP hydrolysis [54]. Al toxicity also correlates with increased lipid and copper transporters, affecting ROS homeostasis and gene expression related to malondialdehyde and protein carbonyl content [55].

## 5. Root Phenotypes and Quantitative Trait Loci for Aluminum Toxicity

Soil acidification, particularly from ammonium fertilizer use, has been a major issue in recent decades. This is especially true in areas with inorganic fertilizers and lowland rice fallows [56]. Studies show that excess Al^3+^ in soil disrupts root growth, primarily by hindering microtubule formation in root hairs. Therefore, tolerant cultivars should possess several quantitative trait loci (QTLs) that enable the sensing of soil acidity, the induction of receptor proteins on cell membranes, the perception of Al-induced redox changes, and the amplification of antioxidation pathways [57]. Major QTLs related to Al toxicity might also be linked to water or other metal stresses, often involving channel proteins, gateway proteins, and other transmembrane domains [58].

Other vital QTLs include transcription factors for genes of cell wall modifications, such as pectin methyl esterase. These genes, upregulated within six hours, help form stable complexes with Al. Their enzyme activity also relates to the metal’s adsorption capacity, suggesting bio-sequestering [59]. Al stress remodels cell wall structures, involving sugar residues and special QTLs with glucosyl transferase on the cell wall, impacting not only glucose but also galactose, glucuronic acid, and xylose modifications [60,61].

Recent developments have introduced strategies to modulate complex interactions of environmental stresses, including the use of bio-stimulants to modify root growth under Al toxicity [62]. For instance, pyroligneous acid, a common bio-stimulant, can reduce Al-induced root growth inhibition, enhancing resilience to metal toxicity and restoring yield [63]. This compound, derived from carbonated low-oxygen conditions, is a mix of bioactive water-soluble moieties, such as sugar and alcohol derivatives, and phenolics and organic acids.

In crops like *Triticum*, certain QTLs, like those for anaerobic carbohydrate metabolism (alcohol dehydrogenase, lactate dehydrogenase, pyruvate decarboxylase), have been identified for Al toxicity resistance [64]. Bio-stimulants can also enhance antioxidative enzymes like peroxidases and promote transcription factors (auxin response factor) in seedlings pre-treated for Al toxicity. These QTLs link carbon concentration mechanisms to Al toxicity, focusing on the development of primary metabolites like organic acids and glucosyl residues [65]. Al tolerance also benefits plants through improved photosynthetic efficiency, as well as the acquisition of reserve carbohydrates, particularly in C_4_ plants.

## 6. Metabolite Shifting from Central Carbon Metabolism

Plants have adapted cellular and metabolic strategies to mitigate Al toxicity. Typically, the citric acid pool and other organic acids form complexes with Al, aiding in its exclusion [66]. This mechanism involves slow Al entry into root cells, followed by acid secretion, acid ionization, and the formation of chelate compounds with Al at the center [67]. In the rhizosphere, plants secrete malic acids and siderophores to chelate metals in extracellular spaces [68]. Tolerant cultivars are capable of bio-exclusion of intracellular Al by forming non-toxic aluminum–chlorohydrate (Al-COOH) complexes. This also complements enhanced carbon concentration mechanisms.

In both C_4_ and C_3_ species, stable carboxylated products like 3-phosphoglyceraldehyde are crucial for Al interaction [69]. Central carbon metabolism supplies carbon intermediates for osmolytes, antioxidants, and other secondary metabolites aiding in Al tolerance [70]. The Calvin cycle, glycolysis, hexose monophosphate shunt, and the citric acid cycle contribute to respiratory substrates, growth, and yield under stress, requiring substantial adenosine triphosphate (ATP) consumption [71]. Studies indicate metabolic shifts from primary metabolism to transitory pathways with specialized metabolites, particularly shikimic acid pathway-derived flavonoids in roots [72].

Mitochondrial metabolism of pyruvate, involving oxidative decarboxylation and oxidative phosphorylation, is enhanced for ATP biosynthesis in response to Al influx in roots. Glycolytic and other anaplerotic reactions produce pyruvate, influencing downstream organic acid pathways under the control of respiratory flux in mitochondria. These acids, particularly malic and citric, can lower intracellular and rhizospheric pH, reducing Al solubility [73]. Enhanced photosynthetic activity in Al-tolerant cultivars is linked to these downstream residues from the central carbon pool [74].

Identifying specific root metabolomes and shifts in other reactions are essential for sustaining photosynthesis and respiration in tolerant species to Al. Recent advancements in omics, especially metabolomics as well as proteomics, offer identifications of complex reaction webs under Al stress [75]. Analyzing qualitative and quantitative aspects of Al-induced metabolomes helps in understanding signal transduction and genome alteration, with rice varieties serving as effective models for studying root-based metabolite changes [76]. Both constitutive (*ALMT* families) and inductive (*MATE* families) genes for metabolomes play significant roles in Al tolerance, whether through exclusion or chemical detoxification.

## 7. Aluminum-Induced Signaling for Reactive Oxygen Species Development

Al toxicity is most severe in its soluble form, particularly as free Al^3+^, which is prevalent in acidic environments as aluminum hydroxide ions [Al(OH)^+^ and Al(OH)^2+^] [77]. Al-induced phytotoxicity primarily starts from specific ion effects, leading to ROS generation and various cellular functions. ROS generation often results in programmed cell death (PCD) characterized by caspase-like activation, chromatin condensation, nuclear dehydration, and chromatin fragmentation [78]. Al tolerance in plants involves successful regulation of cellular redox, enabling root growth even with significant metal accumulation [79]. This tolerance is a genotypic trait linked to antiapoptotic processes that balance cellular redox.

In mitochondria, tolerant species regulate mitochondria-dependent PCD, including cytochrome c release and caspase protease activation [80]. ROS generation in plants under Al stress is a key factor in oxidative damage, similar to that observed under biotic and abiotic stress [81]. Al toxicity triggers oxidative bursts in chloroplast and mitochondrial compartments, disrupting normal redox reactions [82]. Although Al is not a transition metal and does not catalyze redox reactions like Fenton’s reaction, it still induces ROS formation in mitochondria. This is facilitated through processes like the univalent reduction of O_2_ [83]. Subsequent reactions for the reduction of superoxide into H_2_O_2_ are cytotoxic, but also play a dual role in signaling for acclimation and tolerance, yet at low concentrations. Thus, higher H_2_O_2_ concentrations accelerate PCD and senescence.

The relationship between Al toxicity, ROS bursts, and antioxidative enzyme synthesis is crucial for understanding plant survival under oxidative stress [84]. This understanding can help mitigate lipid peroxidation in cellular membranes and compartments. Current research focuses on the correlation between Al toxicity and plant survival in acidic soils, where Al^3+^ bioaccumulation is a concern. Agronomic measures to neutralize soil acidity, such as the use of amending chemical residues, are essential for managing this issue.

## 8. Comprehensive Genomics for Aluminum Toxicity

Al tolerance in different species, including *Oryza sativa* L., which is more tolerant than other cereals like maize, rye, sorghum, and wheat, involves varied gene expressions in roots against the metal [20]. This includes specific Al-responsive gene expressions involving transcription factors (TFs), particularly basic leucine zipper (*bZip*) and regulatory enzymes like protein kinases [85]. Al toxicity is also understood to involve post-translational modifications such as phosphorylation and adenylation of TFs, which are responsible for gene functioning [86]. This mechanism includes the repression, activation, and co-regulation of genes related to metal tolerance.

Al signaling involves different chemical species interactions with root proteomes, leading to changes in gene expression and root activities [87]. Molecular studies in rice have identified and cloned several QTLs like signal transduction and activation of RNA 1, Nramp family 1, *Oryza sativa* acetolactate synthase 1, and *Oryza sativa* magnesium transporter 1 (*STAR2*, *Nrat1*, *OsAlS1*, and *OsMGT1*), which are significant in the context of Al ion (Al^3+^) tolerance [88]. These genes are involved in the generation of superoxides, free radicals, and other ROS-producing genes.

In *Arabidopsis*, salicylic acid-induced nicotinamide adenine dinucleotide phosphate hydrogen [NADP(H)] oxidase reactions are common for specific ROS production, regulating Al toxicity metabolomes [89]. Al^3+^ and other ions initially induce nitrogen oxidase (NOX) activity, linked to superoxide production. Two pore segment channel 1 (TPC1) channel protein activation on the membrane increases Ca^2+^ influx [90]. This influx triggers salicylic acid synthesis and accumulation, which in turn boosts NADP(H) production in root tissues, affecting cellular redox [91].

Vacuole-mediated cell death, a genome-responsive pathway for Al toxicity, is another mechanism, differing from mitochondria-associated cellular death where redox-responsive genes are crucial [92,93]. This process, regulated by proteolytic genes, leads to vacuole collapse and membrane disruption in roots, especially under Al exposure [94]. Protease activity, NADP(H) oxidase, and TPC1 activation are considered key paths for Al resistance [95].

Al phytotoxicity also involves vacuolar membrane lysis, leading to vacuole collapse. In tissues with excessive Al, caspase-like activities trigger vacuolar processing enzyme (VPE) functioning, disrupting cell membranes. VPE functions are also linked to root growth sensitivity, particularly in meristematic regions sensitive to Al toxicity. Numerous genes including VPE1 QTLs are upregulated in response to Al toxicity, indicating their involvement in cell death events [96].

## 9. Special Metabolomic Pathways to Register Aluminum Toxicity

Mitochondrial pathways in response to Al toxicity involve a specific branch point at the ubiquinone (ubQ) site, triggering alternative pathways. The upregulation of genes related to the vacuolar processing enzyme 1 (VPE1) QTL has been linked to cell death events under Al toxicity. Alternative oxidase (AOX) diverts electrons from the main transport chain to reduce molecular oxygen without energy production, thereby reducing reactive oxygen species (ROS) burden under Al toxicity [97]. Excessive Al causes complex II and III to be bypassed, leading to a reduced ubQ pool, indicating oxidative redox activity [98].

**Figure 5:**
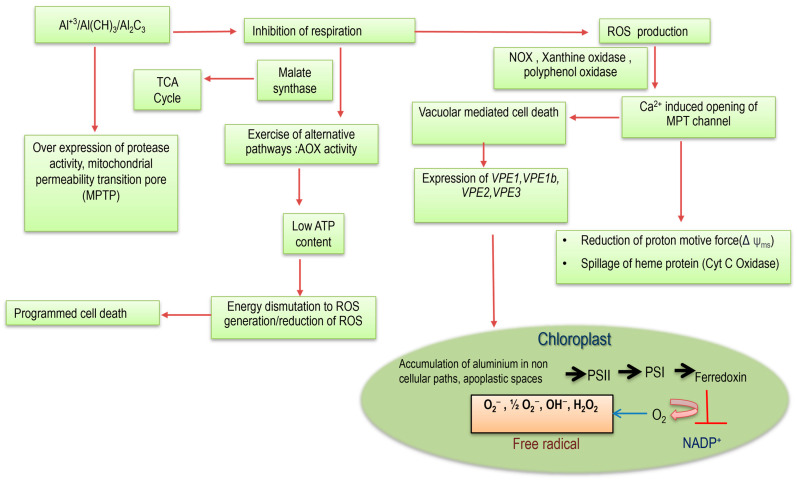
Depicting the genotoxic effects of Al through various intermediate gene expressions that individually or synergistically influence cellular phenomena. Initially, Al^3+^ stimulates ATP production, while simultaneously downregulating the expression of other electron transport carriers, leading to the dismutation of oxygen into free radical ROS. This ROS production triggers the tonoplast membrane, where Ca^2+^ activates mitochondrial permeability transition (MPT) channels. Concurrently, vacuole-mediated cell death is facilitated by the overexpression of vacuole-localized cysteine protease genes like *vacuolar processing enzyme* genes (*VPE1*, *VPE1b*, *VPE2*, *VPE3*), releasing Al from the vacuole into the cytosol. The over-activation of MPT channels disrupts the proton motive force (∆ψ_m_) and causes spillage of cytochrome c oxidase, underlying the oxidative damage. The TCA cycle is activated, triggering anaplerotic reactions and inducing genes for malate synthase, citrate synthase, and oxaloacetate decarboxylase. Mitochondrial membrane leakage, linked to the overexpression of mitochondrial membrane permeability transition pore (MPTP) proteins and increased protease activity, also contributes to this process. Collectively, these events culminate in programmed cell death (PCD) of the roots, completing the cycle of Al toxicity.

Under high Al toxicity, there is a significant increase in alternative oxidase (AOX) transcripts, regulated both transcriptionally and translationally. In non-stressed plants, both transcript abundance and enzyme activity of AOX are low. Al accumulation downregulates root respiration through complexes III and IV, diminishes succinate-dependent electron transfer, and reduces cytochrome oxidase activity [99]. The results of increased ROS and free radicals can consume energy to reduce ROS with the participation of nicotinamide adenine dinucleotide phosphate NADP(H), flavin adenine dinucleotide (FADH_2_), and other reducing equivalents. Plant response to Al is finally accomplished at the nuclear level where a number of gene expressions ensure various intermediate products that influence the cellular phenomena either singly or in combination (Figure 5).

Differential gene expression shows that major TCA cycle enzymes like succinate dehydrogenase, alpha-ketoglutarate dehydrogenase, and malate synthase are targeted to generate NADP(H), bypassing complexes III and IV to AOX [100]. Al-treated tobacco roots experience a significant increase in ROS and a consequent decline in ATP synthesis [101]. This suggests that Al-induced inhibition of respiration downregulates mitochondrial ATP production, affects the bioenergetics status of tissues, and potentially induces the signaling of programmed cell death (PCD) in roots. This effect may extend to shoot tissues, where Al toxicity leads to early senescence and foliage abscission [102].

Furthermore, the mitochondrial genome under Al toxicity overexpresses special proteins on the outer membrane. Excess Al toxicity opens mitochondrial transition pores, leading to the leakage of matrix-based proteins, loss of matrix potential (ΔΨm), and impaired ATP generation [103]. Al toxicity stimulates these pores, releasing cytochrome c oxidase proteins, ultimately triggering PCD [104]. Oxidative stress, therefore, is linked to mitochondrial metabolomics, potentially driven by elevated Ca^2+^ concentration and increased ROS, leading to further peroxidation reactions. Mitochondrial permeability transition, influenced by Ca^2+^ under Al toxicity, involves specific membrane proteins in the process of cell death.

## 10. Special Metabolites and Their Contribution to Al Tolerance in Plants

Tobacco cells exposed to Al toxicity accumulate caffeic acid and chlorogenic acids via the phenylpropanoid pathway. A key enzyme in this process is L-phenylalanine ammonia-lyase (PAL), whose activity is linked to phosphate availability in plant tissues. Aluminum toxicity induces phosphate deprivation, increasing PAL activity [105]. The phenolics produced by this pathway act as antioxidants, protecting against lipid and protein peroxidation and macromolecule carbonylation. When exposed to a combination of Al^3+^ and Fe^2+^, plant cells activate these antioxidants, indicating tolerance against this joint combination of metals. This suggests that the combined effects of metals are mediated by the induction of phenolic antioxidants like phenylpropanoid residues and supersedes the individual effects of ions [106].

Organic acids from the TCA cycle are other metabolites reducing rhizotoxicity in Al-contaminated soil. Al ions (Al^3+^), prevalent in acidic soils, induce proton (H^+^) secretion in some sensitive species. The acidic pH, with its high H^+^ concentration, enhances solubilization in the soil, mitigating phytotoxic impacts. A lower pH can also reduce the negative charge of biological membranes, decreasing Al binding affinity [107].

In *Arabidopsis*, citrate induction is crucial for the expression of the transcription factor *Arabidopsis thaliana* SENSITIVE TO PROTON RHIXOTOXICITY 1 gene (*AtSTOP1*), associated with specific H^+^ transporters. Mutations in STOP1 can reduce Al tolerance due to inadequate citrate production, indicating *AtSTOP1’*s role in activating other genes, including those in antioxidative cascades, for co-tolerance against Al^3+^.

Other metabolites include methylated residues formed during reactions to Al toxicity, contributing to H^+^ de-acidification. A protein similar to glycerophosphodiesterase (GPD) is overexpressed at the transcript level in tobacco soon after Al exposure, which is necessary for the methylation of CCGG islands in the promoter regions. This methylation pattern changes with Al exposure, similar to the response to herbicide-induced ROS [108]. Since ROS are common in metal induction, demethylation may occur through the development of oxygen radicals. Cytosine residue methylation decreases upon Al^3+^ application in roots, indicating that metal induction can cause total DNA methylation, potentially leading to genotoxicity.

## 11. Metabolomes under Regulation of Signal Transduction and Protein Turnover

Several metabolic residues are believed to be involved in signaling and perception pathways through the cell membrane during Al transportation. Specific guanosine triphosphate (GTP) binding proteins, isolated from cell membranes, are known to function in ion channel opening, cell volume proliferation, and energy-mediated processes [109]. Various species, including cereals and *Arabidopsis*, show changes in mass proteome expression in roots exposed to Al, indicating alterations of global gene expression related to antioxidation. Vacuolar hydrogen ion ATPase (H^+^/ATPase) activity in roots is suggested to create an electrochemical gradient for Al^3+^ activation through a proton antiporter system [110]. In Al-tolerant *Triticum* species, a tonoplast ATPase protein is overexpressed, linked to metal absorption, though reports vary on ATPase expression levels across different crop species irrespective of Al sensitivity and tolerance. This suggests that Al compartmentalization is an avoidance strategy, with plants variably responding to H^+^/ATPase proteome expression for exclusion [111].

Mitochondrial ATPase activity, isolated from mitochondrial inter-membrane space, varies with protein subunit expression. For instance, soybean shows reduced membrane-bound ATPase activity but increased mitochondrial ATPase under Al toxicity. Parallel expression of organic acid-secreting proteins and P-type ATPase suggests dual roles in sequestering Al in vacuoles or apoplastic spaces following esterification. In legumes like soybean, an increase in citrate synthase activity from the TCA cycle correlates with co-transport by ATPase-driven activity.

Homeo-domain proteins from the MCM1, AGAMOUS, DEFICIENS and SRF-Box (MADS-Box) family in soybean have distinct DNA binding domains for regulatory factors in Al tolerance genes [112]. Metabolomes contributing anaplerotic reactions with citrate, malate, and oxaloacetate are crucial for chelating metals in apoplastic spaces. In some rice cultivars, tolerance to Al is linked to overexpression of MADS-Box factors, enhancing expression of *vacuolar processing enzyme* (*VPE*) genes and H^+^ extrusion [113].

Protein regulation through lysis is also significant in Al toxicity, where root growth retardation is linked to protein lysis [114]. In rice and other crops, the induction of proteasomes and heat shock proteins (HSPs) occurs under varying Al^3+^ concentrations where HSPs are involved in protein folding, transport, stress resistance, and gene expression [115]. This suggests the necessity of protein post-translational modification under Al toxicity. The induction of HSP70 and its derivatives might be a key factor in Al tolerance, with protease activity indicating tolerance through the turnover of degenerative proteins. In tolerant cultivars, protease-mediated lysis of misfolded proteins may alleviate degradation from root membrane lysis and growth inhibition [116].

## 12. Transcriptional Control of Aluminum Tolerance in Roots under Acidic Cytosol

An acidic environment is the most favorable key to Al sensitivity in root physiology, where a number of gene activations are directly or indirectly involved through transcription factors. Transcription factors have been known of for the last few decades with regard to plant responses to different stimuli. Factors like ADP-ribosyltransferase 1 (*ART1*) in rice are most important for Al tolerance, as already mentioned earlier [117]. Now, there is a quest to identify a few interacting proteins with *ART1*. Already, nine of the downstream genes induced by *ART1* factors have been functionally characterized for Al tolerance exclusively in rice [118]. Moreover, tolerant species to Al have also been scanned for a few QTLs and used as molecular markers. Still, the fine mapping and cloning of these QTLs are yet to be deciphered for breeding. Acidic soil is a major problem for cultivated crops in which transcriptome-wide association studies combined with genome-wide association studies are required to identify the direct involvement of gene versus trait association. In addition, for the well-characterized *ART1*-regulated gene, the membrane localized polypeptides of 53 amino acid residues are identified [109]. The gene *C-terminal domain phosphatase* (*CTD3*) is essentially metal-specific and exhibits no transport ability for Al but directly binds to Al in roots [119]. Moreover, *CTD3* from rice (*OsCTD3*) is specifically required with a strong promoter for its expression on membranes to detoxify Al. External detoxification in roots for Al also involves a lot of acid secretion in the rhizosphere rather than internal secretion of the metal. In a number of cases, biomolecules like pectin, hemicelluloses, and other polysaccharides on the root symplast, as well as endodermal casparian strips, are required for sequestration of the metal [120].

**Figure 6:**
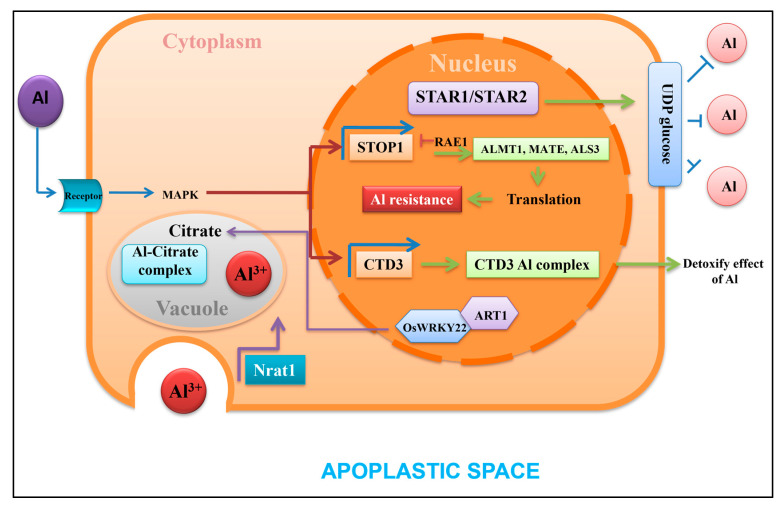
A hypothetical model for control of organic acid (citrate) exudation in roots against Al ion (Al^3+^) toxicity. At the nuclear level, activation of SENSITIVE TO PROTON RHIXOTOXICITY 1 (STOP1) initiates the signaling pathway, which otherwise activates *multidrug and toxic compound extrusion 1*, *Arabidopsis thaliana aluminum activated malate transporter 1*, and *amylotrophic lateral sclerosis 3* (*AIMTE*, *MATE*, *ALS3*) genes. STOP1 also upregulates the expression of *RNA export 1* (*RAE1*), which, in feedback regulation, reduces STOP1 through degradation and ubiquitination. *Signal transduction and activation of RNA 1 and 2* (*STAR1*, *STAR2*) otherwise induce UDP glucose-like residues, which conjugate with Al for extracellular detoxification. Transcription factors like *Oryza sativa* wrinkled transcription factor (*Os*WRKY22) form dimers with ADP-ribosyltransferase 1 (ART1) and collectively induce citrate biosynthesizing genes. Expressed proteins from the Nramp family (Nrat1) on the cellular membrane engulf Al^3+^ within the apoplastic space for chelation.

The modality of gene regulation with transcription factors through phosphorylation–dephosphorylation and adenylation–deadenylation is important for cell signaling with Al. In acidic soil, a C_2_H_2_ zinc-finger protein homologue to SENSITIVE TO PROTON RHIXOTOXICITY 1 (STOP1), as found in *Arabidopsis,* can regulate malate transporters *Arabidopsis thaliana* multidrug and toxic compound extrusion 1 and *Arabidopsis thaliana* aluminum activated malate transporter 1 genes (*AtMATE1*, *AtALMT1*). These two factors can bind the basic upstream elements [GGN(T/g/a/C)V(C/A/g)S(C/G)] where the downstream genes would be malate, citrate, oxalate, and other organic acids [121]. This promoter can control a minimum of 30 genes covering *expansin A10*, *magnesium transporter 1*, *signal transduction and activation of RNA 1*, and *ferric reductase defective like 4/2*, (*EXPA10*, *MGT1*, *STAR1/2*, *FRDL4*, *FRDL2*), expressed in extracellular or intracellular spaces [122]. The specific ATP-binding cassettes and membrane binding domain, similar to *E. coli* ABC transporters, highlights that the two basic transcription factors (STAR1-2) of the *ALMT1* gene are more characterized. The homeodomain complex STAR1-STAR2, localized at the membrane, transports pyrimidine nucleotide (UDP-glucose), which is required for the modification of the cell wall and callose [123]. Transcription factors like FRDL4 are required for the secretion of organic acids under Al toxicity in roots for internal detoxification. These factors include (cys-cys)_n_, a homologue peptide that can directly bind with Al and reduce Al detoxification, although externally. On the other hand, magnesium transport and upregulation of *MGT1* can reduce internal Al concentration, superseding other metals [124]. For the vacuolar sequestration of Al, other members of Nramp gene family 1 (*Nrat1*) are important, specifically for aluminum ions (Al^3+^). For the same species, a tonoplast-bound ABC transporter is recognized from rice, *Oryza sativa acetolactate synthase 1* (*OsALS1*), which takes part in the activation of promoters under low pH. There exists a strong correlation for Al tolerance with the expression of *OsALS1* and *OsFRDL4*-like factors in roots under acidic pH of soil [125]. Along with the normal ability in organic acid transportation, genes encoding expansion-like proteins for cell wall elongation in root tips are dependent on the activation of transcription factors. From the most sensitive cereals to Al (e.g., rice), another few transcription factors like ART2 are also important to interact with other factors (Figure 6). Likewise, wrinkled transcription factor (WRKY) domains, which are characterized by a zinc-finger structure (either Cx4-5Cx22-23HxH or Cx7Cx23HxC) on the upstream sequence containing W-BOX promoter, are also applicable for Al sensitivity [126]. Many factors against Al toxicity like STAR1/2 can interact with WRKY in a dimer configuration, where the *cis*-element is represented with a consensus sequence of TTGACC/T [127]. The expression of *OsFRDL4* is controlled by *Os*WRKY22, where the localization of expression is the plasma membrane, nucleus, tonoplast, etc. Sometimes, co-expression of *OsWRKY22* and *ART1* is found in dimer configuration within the nucleus for citrate secretion. A few factors like abscisic acid stress and ripening (*ASR*) genes are identified in response to wider activation of Al-tolerant genes. These ASR factors mostly act as chaperones and transcription factors and are amenable to the *ASR5* gene, involved in Al tolerance in rice [128]. *ASR5* is nucleus- and cytoplasm-specific in expression and regulates the co-expression of different genes from Al-induced stress tolerance. In more recent versions of genome-wide arrays of activating genes, the ASR5 *trans*-factor is shown to bind the STAR1 promoter and, downstream, can also regulate *Nrat1*. Also from rice, *ALS1* encodes an ABC transporter belonging to transporter-associated antigen processing protein (TAP), which can be induced only by Al^3+^ but not other metals at low pH [129]. These are further supported by the expression of transcription factors for the genes expressed in rice—*Oryza sativa pectin methylesterase* (*OsPME*)—required for pectin methyl esterase in Al detoxification for apoplastic spaces. Conclusively, transcriptomes for Al tolerance are mostly based on the sensitization of transcription factors where proteins, either singly or in combination, can regulate the expression where molecular mechanisms are concerned.

## 13. Phytohormone Signaling and Functioning for Aluminum Tolerance

**Figure 7:**
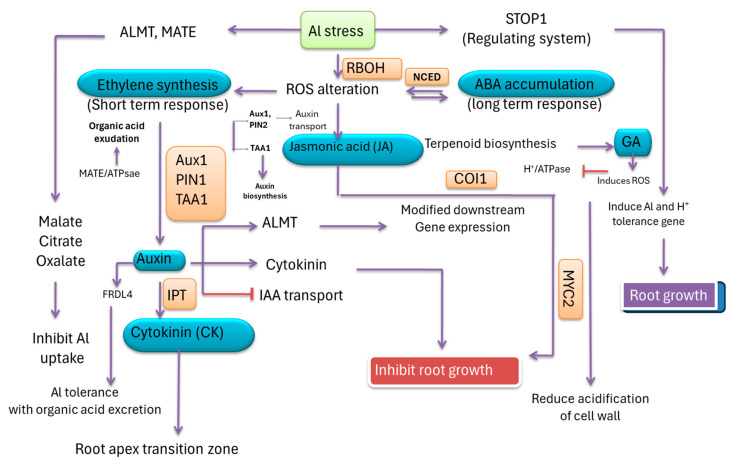
The involvement and crosstalk of plant growth substances against Al sensitivity in plants. Growth hormones like Aux, GA, ABA, CK, JA, and ET induce several cellular processes in roots of plants exposed to Al hyper-accumulation. Both external and internal detoxification by exclusion, sequestering, and chelation reactions are important under hormonal influence. Reduction of H^+^ by inhibition of H^+^/ATPase activity is important to downregulate the acidification of apoplasts of roots to reduce Al ions (Al^3+^). ET induces auxin accumulation as well as auxin transportation by pin formed 2 (PIN2)-mediated polar transport that results in root growth inhibition. Acidification of cell wall by organic acids (malate, citrate, oxalate) is important for ligand formation with metal. ABA and ET have synergistic actions with negative regulation, where RBOH and short-term response, respectively, are important. ABA can also induce ROS biosynthesis, which directly influences Al tolerance genes. Other substances like JA and CK are distantly related to Al tolerance by overexpression of *coronatine insensitive 1* and *myeloblastosis viral oncogene homologue 2* (*COI1*, *MYC2*) and direct inhibition of root growth.

Al undoubtedly invites metal stress and is also coincided by plant hormones including auxin (Aux), gibberellins (GA), ethylene (ET), abscisic acid (ABA), jasmonic acid (JA), and cytokinin (CK), as well as other secondary elicitors like nitric oxide [130]. External applications of plant growth regulators and their synthetic analogs, in most cases, record similar effects. Rice under acidic soil shows a strong affinity for Al^3+^ sensitivity and thereby induces specific transcription factors like ADP-Ribosyltransferase 1 (ART1), a C_2_H_2_ zinc-finger motif. This factor specifically plays a role in the regulation of 31 downstream genes under Aux control [131]. The genes include rice aluminum transporters like nicotinate riboside transporter 1*/Oryza sativa* nicotinate riboside transporter 4 (*NRAT1/OsNRAMP4*), which are characteristically Al tolerance genes where Aux metabolism is involved. Since root growth is the most vulnerable to Al toxicity due to inhibition, changes in Aux synthesis and translocation may be important, as recorded in *Brassica* [132]. Aux signaling, with its principle function being stress-induced root growth inhibition, has demonstrated the expression of Aux transporter proteins like *Oryza sativa* pin formed 3 (*Os*PIN3t). This transporter activity is also observed with the reduction in adventitious growth in *Arabidopsis*. The toxicity of Al inhibits the expression of the *pin formed 2* (*PIN2*) gene encoding protein for auxin transport towards the roots. Downregulation of its expression can reduce endogenous Aux concentrations in roots, and thereby, sensitivity to Al is more marked, impeding root growth [133]. Other transporter genes for Aux, including *PIN2*, *ferric reductase defective like 4* (*FRDL4*), and *auxin resistant 1* (*AUX1*), are also proposed to correlate with ethylene production. ET undoubtedly happens to be a good signaling residue, resulting in impeded root growth under Al toxicity [134]. ET has a direct relationship with Al^3+^ stress, where RBOH-mediated ROS alteration can induce its synthesis. ET is also a direct signaling residue for auxin biosynthesis, where genes like *Aux1*, *PIN1*, and *tryptophan aminotransferase related 1* (*TAA1*) are involved in root growth inhibition under Al stress [135]. ABA is typically recognized as a stress hormone, also cloned from rice, where a few homologues of the abscisic acid-stress-ripening (*ASR*) family are demonstrated under ABA and Al^3+^ stress simultaneously. The increase in synthesized ABA is directly related to cellular signaling, where H_2_O_2_ is the most important ROS. The signaling cascade under Al stress, as perceived through cell membrane-bound receptors, is also co-induced with *9-cis-epoxycarotinoid dioxygenase* (*NCED*). This gene encodes the protein that splits the carotenoids for ABA biosynthesis. Higher expression of *NCED* accumulates intracellular ABA in support of osmotic adjustment under Al^3+^-stressed crops [136]. ABA synthesis in Al-treated root cells can induce ATP-binding cassette transporter (ABC) and nitrate transporter1/peptide transporter (NPF) for its translocation in different organs. Subsequently, ABA serves as a stimulus for the resistance mechanism against Al toxicity by increasing water use efficiency and decreasing transpiration rates [137]. Additionally, ABA accumulation in some root tissues induces the formation of a protein complex via receptors like pyrabactin resistance 1/pyrabactin resistance-like (PYR/PYL) and protein phosphatase 2C (PP2C). This complex induces the activation of some protein kinases which otherwise activate by the phosphorylation of specific anion channels to release malate against Al accumulation [138]. This family encodes specific transcription factors such as amino-terminal enhancer of split-related 5 (*Os*AsR5), responsible for the co-expression of *signal transduction and activation of RNA 1* (*START1*) in the Nramp family of factors [139]. Another gene, *FRDL4,* has also been cloned from roots sensitive to Al^3+,^ where the tolerance to the metal is manifested by the development of ROS. The ROS would otherwise be an inducer for different genes related to Al tolerance, more specifically the synthesis of organic acids. The organic acids in turn are also induced by some growth regulators like ethylene under Al stress [140]. ET in wheat has also been reported with negative control for *Al activated malate transporter* (*ALMT1*) that reduces the root secretion of organic acids. Thereby, mutation of this gene would be sufficient for sensitivity to Al toxicity [141]. ET has also been reported for Al tolerance, yet in crosstalk with Aux interference. The expression of ethylene biosynthetic genes like *1-aminocyclopropane-1-carboxylic acid synthase* (*ACSs*) and *1-aminocyclopropane-1-carboxylic acid oxidase* (*ACOs*) induces a direct promotion of ethylene biosynthesis under Al^3+^ toxicity in sensitive cultivars [142]. On signaling pathways, ET activates the expression of transcription factors like ethylene insensitive 3 and 1 (EIN3 and EIN1), which otherwise control root growth. EIN3 has a good binding ability to the promoters of a few genes (*YUCCA 9, YUC9*) to interfere with the growth of the root apex transition zone under Al toxicity [143]. The toxicity of Al in root sensitization finds the expression of a few genes (*TAA1*, *YUCs* [*YUC3*/*5*/*7*/*8*/*9*]) for transcription factors in the regulation of ethylene biosynthesis in contrast to Aux. Therefore, the toxicity of Al in root growth moderation would be a supplementary effect, where Aux and ET expression in effected tissues is simultaneous. ET is also responsible for the regulation of both local Aux biosynthesis and Aux transport by the induction of genes like *TAA1*/*YUCs* and *PIN2*, and *AUX1,* respectively [144]. The distinct action of ET in the reversal of Aux sensitivity is variable through plant species when exposed to Al^3+^; still, their molecular mechanisms are obscure. GAs are implicated in metal tolerance through the preservation of carbohydrate metabolism and vegetative growth. In that context, Al^3+^-induced inhibitions of root elongation following sugar translocation to roots are also reported as possible pathways for metal tolerance. Still, downregulation of Al-induced ROS accumulation and changes in root cell wall deposition may be apprehended as membrane-localized Al sequestering [145]. The function of GAs with multiple attributes may collectively include the biosynthesis of bioactive GAs in tomatoes, improving growth and pigment content, as well as the CO_2_ fixation rate in soybean under metal stress [146]. CT is less explored under Al toxicity but is also related to cell wall alkalization, an influx of H^+^ over the membrane, and, in a few cases, alteration of expression for cell wall modifying genes. CT biosynthesis is directly influenced by Aux mediation, where transcription factors like transport inhibitor response 1 (TIR1) and auxin signaling f-box (AFB) are responsible for encoding other factors on upstream binding sequences. These factors most importantly include auxin response factors (ARFs) 7–19, which are otherwise involved in isopentenyl pyrophosphate (IPT) residue biosynthesis as a precursor of CT [147]. Growth substances like JA also play a differential role against Al toxicity. JA application in Al-sensitive root species induces adaptive responses in plants by the regulation of ROS metabolism (Figure 7). Two genes, *coronatine insensitive 1* (*COI1*) and *myeloblastosis viral oncogene homologue 2* (*MYC2*), are found to be overexpressed in root growth inhibition by altering the ET concentration. Phenotypic analysis with mutants insensitive to JA biosynthesis and signaling was recorded in response to Al^3+^ with direct involvement for ET accumulation. The inhibition of formation and depolymerization of cortical microtubules in those mutants are responsible for *COI1* gene expression under Al stress [148]. Moreover, malate transporter expression by aluminum activated malate transporter (*ALMT*) is also dependent on the regulation of JA signaling where *COI1* is involved. Therefore, phytohormones, a crucial signaling residue in favor of Al tolerance, play pivotal roles, mostly in metal sequestering, ion complex formation, water balance, and ROS homeostasis.

## 14. Conclusions and Future Perspectives

This discussion highlights that Al toxicity impacts plants in two main phases: the establishment of metal-induced specific ion effects and the development of an oxidizing redox environment prone to peroxidation reactions. Initially, physiological and cellular events are affected, including reduced root membrane permeability, increased dehydration, vacuolar disintegration, nutrient release over the tonoplast membrane, cytosolic pH alterations, caspase activity acceleration leading to PCD, and dissolution of cellular metabolites into soluble products. Conversely, oxidative redox changes can lead to biomolecule degradation, including lipid peroxidation, protein carbonylation, and demethylation of specific coding regions or gene promoters. Altered metabolite fluxes, particularly in organic acid turnover, can enhance Al toxicity tolerance. Another key tolerance mechanism is the pattern change in secondary metabolites, especially the development of complex polysaccharides like callose, preferred in selective cultivars. This development is also linked to alternative oxidase (AOX) expression and ATPase activities, which help prevent electron misfiring from cellular organelles and stimulate energy-producing pathways. Additionally, specialized transcriptional regulation by MCM1, AGAMOUS, DEFICIENS, and SRF-Box (MADS Box) proteins and other transcription factors is crucial for activating specific genes under Al toxicity. This process is complemented by protein degradation mechanisms that impede root growth, countered by the expression of heat shock proteins (HSPs) or chaperones, which aid in refolding misfolded proteins. Therefore, Al tolerance is a multifaceted process involving both enzymatic antioxidation cascades and bio-exclusion of the metal through diverse gene expressions. Future prospects include profiling different proteomes in Al-tolerant species to understand and manage various pathways under Al stress. For instance, examining proteomes involved in cysteine and methionine metabolism could shed light on methyl cycling and glutathione metabolism for antioxidation. Recent advances in proteomic sequencing are poised to provide deeper insights into precise protein expression and its role in Al tolerance. Over the past few decades, people have been on a quest to develop strategies for crops under Al contamination. Many aspects of Al cytotoxicity remain uncovered and could be targeted along with suitable screening methods for crop species that accumulate less Al. In sustainable breeding, further identification of specific QTLs governing Al tolerance should be the focus area. Along with the advent of physical technologies that are useful in environmental decontamination and the restoration of soil fertility, the development of Al-resilient crops would be a pathway for food security against acidic soil in countries in the Global South.

## Data Availability

All information is available in this article.
